# Lessons learned from rapid exome sequencing for 575 critically ill patients across the broad spectrum of rare disease

**DOI:** 10.3389/fgene.2023.1304520

**Published:** 2024-01-08

**Authors:** Abderrahim Marouane, Kornelia Neveling, A. Chantal Deden, Simone van den Heuvel, Dimitra Zafeiropoulou, Steven Castelein, Frank van de Veerdonk, David A. Koolen, Annet Simons, Richard Rodenburg, Dineke Westra, Arjen R. Mensenkamp, Nicole de Leeuw, Marjolijn Ligtenberg, Rene Matthijsse, Rolph Pfundt, Erik Jan Kamsteeg, Han G. Brunner, Christian Gilissen, Ilse Feenstra, Willem P. de Boode, Helger G. Yntema, Wendy A. G. van Zelst-Stams, Marcel Nelen, Lisenka E. L. M. Vissers

**Affiliations:** ^1^ Department of Human Genetics, Radboud University Medical Center, Nijmegen, Netherlands; ^2^ Department of Neonatology, Radboud University Medical Center, Radboud Institute for Health Sciences, Amalia Children’s Hospital, Nijmegen, Netherlands; ^3^ Research Institute for Medical Innovation, Radboud University Medical Center, Nijmegen, Netherlands; ^4^ Department of Internal Medicine, Radboud University Medical Center, Radboud Institute for Health Sciences, Nijmegen, Netherlands

**Keywords:** rapid exome sequencing, diagnostic workflow, turnaround time, clinical outcome, diagnostic yield

## Abstract

**Introduction:** Rapid exome sequencing (rES) has become the first-choice genetic test for critically ill patients, mostly neonates, young infants, or fetuses in prenatal care, in time-sensitive situations and when it is expected that the genetic test result may guide clinical decision making. The implementation of rES has revolutionized medicine by enabling timely identification of genetic causes for various rare diseases. The utilization of rES has increasingly been recognized as an essential diagnostic tool for the identification of complex and undiagnosed genetic disorders.

**Methods:** We conducted a retrospective evaluation of our experiences with rES performed on 575 critically ill patients from various age groups (prenatal to adulthood), over a four-year period (2016–2019). These patients presented with a wide spectrum of rare diseases, including but not limited to neurological disorders, severe combined immune deficiency, and cancer.

**Results:** During the study period, there was a significant increase in rES referrals, with a rise from a total of two referrals in Q1-2016 to 10 referrals per week in Q4-2019. The median turnaround time (TAT) decreased from 17 to 11 days in the period 2016–2019, with an overall median TAT of 11 days (IQR 8–15 days). The overall diagnostic yield for this cohort was 30.4%, and did not significantly differ between the different age groups (e.g. adults 22.2% vs children 31.0%; *p*-value 0.35). However, variability in yield was observed between clinical entities: craniofacial anomalies yielded 58.3%, while for three clinical entities (severe combined immune deficiency, aneurysm, and hypogonadotropic hypogonadism) no diagnoses were obtained.

**Discussion:** Importantly, whereas clinical significance is often only attributed to a conclusive diagnosis, we also observed impact on clinical decision-making for individuals in whom no genetic diagnosis was established. Hence, our experience shows that rES has an important role for patients of all ages and across the broad spectrum of rare diseases to impact clinical outcomes.

## Introduction

Over the last decade, exome sequencing (ES) has significantly changed the field of medical genetic diagnostic testing without the *a priori* need to know the exact genetic defect, allowing untargeted analysis of all protein-coding sequences in a single testing modality (Vissers et al., 2010; Yang et al., 2013). Recent clinical studies have pioneered with rapid exome sequencing (rES), or even rapid genome sequencing (rGS) approaches for different patient groups, like critically ill neonates admitted to a neonatal intensive care unit or critically ill children later in life, for whom knowledge on the underlying genetic cause would facilitate an individualized management (Stark et al., 2018; Clark et al., 2019; Sanford et al., 2019; Kamolvisit et al., 2021; Krantz et al., 2021; Olde Keizer et al., 2023).

The optimized turnaround time of less than 2 weeks for rES has played a crucial role in its widespread adoption in prenatal diagnostics (Corsten-Janssen et al., 2020; Deden et al., 2020; Faas et al., 2023). The ability to comprehensively assess all known disease-causing genes for various types of variants, including single nucleotide variants (SNVs), small insertion/deletion (indels) events, and copy number variants (CNVs), has quickly established rES as a primary diagnostic test for fetuses with ultrasound-detected (multiple) congenital anomalies, as well as critically ill neonates admitted to a neonatal intensive care unit (NICU) (Olde Keizer et al., 2023; Lord et al., 2019; D’Gama et al., 2022).

Overall, there is a growing awareness of the genetic factors contributing to various diseases observed in patients admitted to intensive care units, encompassing both pediatric and adult populations (Dye et al., 2011; Wu et al., 2019). Combined with the marked reduction in sequencing costs, increased sequencing accuracy and improved variant detection methods, as well as optimized laboratory infrastructure, rES has opened new windows of opportunities for all individuals with a potential rare disease requiring an urgent genetic diagnosis (Kamolvisit et al., 2021). Previous studies on rapid exome testing have mainly focused on fetuses, infants, and children, particularly those in ICU settings. It might be expected that rES is equally useful in older individuals for whom knowledge on the genetic cause of disease may help in medical decision making, such as for instance for choosing specific cancer treatment informed by the underlying genetic cause. There is only limited information on reasons for clinical referral and the overall use of rES in the adult population. We therefore conducted a retrospective analysis of an unselected cohort of 575 individuals who consecutively received rES in our institute between 2016 and 2019. We characterized the diagnostic procedure, including the turnaround time of rES, the diagnostic strategies used and diagnostic yield obtained, and evaluated these parameters across different age groups and the full spectrum of rare disease.

## Materials and methods

### Patients, counseling, and informed consent

Between 01 January 2016 and 01 January 2020, 575 critically ill patients with a suspected genetic disorder received rES. Referral for rES was based on consultation in a multidisciplinary team involving a clinical geneticist and was based on the clinical presentation of the patient, along with the expectation that the obtained genetic diagnosis could help the clinical decision-making process. The clinicians informed the patients (and/or parents) regarding the rES procedure. Written informed consent was obtained for each patient (or legal representative) as well as for all participating relatives. The study was approved by the Medical Research Ethics Committee Arnhem/Nijmegen under file numbers 2016–2486/NL57511.091.16 and 2020-7142.

### Data collection

For each patient, we retrospectively collected all available clinical data. Furthermore, we gathered information regarding the laboratory workflow, such as the laboratory’s receipt date of the specimen, the date of report signing, and the diagnostic gene panel strategy. This encompassed the analysis of the requested *in silico* disease-gene panel(s) and/or Mendeliome. The Mendeliome comprises all genes linked to diseases listed in OMIM.

### Rapid exome sequencing

DNA isolation for postnatal samples (n = 334) was performed from EDTA blood (n = 315) and umbilical cord blood (n = 19) using routine procedures (Diekstra et al., 2015). For prenatal samples (n = 241), DNA was either isolated from chorionic villus cells (n = 25) or amniotic fluid cells (n = 207). DNA from cultured cells (n = 9) was isolated using the Qiagen DNA Mini Kit (Qiagen, Hilden, Germany), whereas DNA from non-cultured cells was isolated using QIAmp MinElute Virus Spin Kit (Qiagen, Hilden, Germany), according to manufacturer’s protocols. For all DNA samples, quality control of the manual process included the analysis of a small set of dedicated SNPs (n = 55) prior to the rES procedure, allowing for comparison at variant level to confirm sample identity after the rES procedure.

DNA concentrations were quantified using the Qubit 3.0 system (dsDNA broad range kit; Invitrogen, Carlsbad, CA). A total amount of 50 ng was taken for enrichment and library preparation, which was performed using the SureSelect Human All Exon V5 exome enrichment baits in combination with the Agilent SureSelect QXT library preparation kit (both Agilent, Santa Clara, CA), according to the manufacturer’s instructions. Samples were barcoded with unique i7 and i5 barcodes. After purification by AmPure beads, the samples were quantified (Qubit 3.0 and Tapestation) for accurate equimolar pooling. The libraries were subsequently sequenced on Illumina NextSeq500 systems (High output, 2 × 151 cycles; Illumina, San Diego, CA), aiming for a minimal exome-wide coverage of 200-fold. After sequencing, the raw data was demultiplexed (bcl2fastq) and aligned to hg19 (BWA, samtools, picard) using a fully automated in-house bioinformatics pipeline which included standardized quality checks including, but not limited to: median insert size (>150 bp); % duplicated mapped reads (<30%); median coverage (>80x); 10x coverage (>90%) and 20x coverage (>80%); per base sequencing error rate (<0.015); % sequence on target (>70%).

Variant specific callers were used including GATK for SNVs, CoNIFER for CNVs and an in house-developed pipeline for Regions of Homozygosity (ROH). For each variant, annotation allowing prioritization was added, including, amongst others, variant effect prediction, population frequencies and previous reports of pathogenicity. In addition, for family-based sequencing, a trio-based analysis was included to allow for prioritization of *de novo* variants. To optimize the processing time, the alignment is run in parallel using FASTQ files per lane, as are calling and annotation for each sample and each caller. Once annotation was completed, an automated message was sent to the laboratory that the sample was ready for interpretation. Also in this part of the pipeline, automated quality control was performed, including variants to be called on all chromosomes; total number of variants (<75,000); number of premature stop codons (<150); Tv/Ti ratio (between 2 and 3); and a biological match for sex chromosomes based on the gender specified in our LIMS system.

### Diagnostic rES interpretation strategies and rES diagnostic outcomes

Variant interpretation of rES was analogous to routine exome sequencing, explained in detail in (17). In brief, interpretation is based on a two-tiered approach. Tier 1 included the analysis of variants restricted to genes known to be associated with the index’s condition by means of an *in silico* gene panel enrichment (Radboudumc, 2023). For instance, an “ID gene panel” could be requested in a prenatal phase based on the clinical observation of structural congenital brain anomalies during a fetal ultrasound imaging, or alternatively, during early childhood when developmental delay was noted. If the patient’s symptoms did not allow for selection of (a) disease-specific gene panel(s), the clinician could also request analysis of the Mendeliome, consisting of 3,839 genes with an OMIM-listed disease-gene association. In case no molecular diagnosis was obtained in tier 1, and the patient consented for further analysis, the analysis was followed by tier 2, allowing for prioritization, interpretation and classification of variants in the Mendeliome (if not already performed in tier 1) and those in genes without known disease-gene associations (so called “open exome analysis”).

During interpretation, SNVs were classified based on a 5-class system (Class 1–5) in accordance with the guidelines from the Association for Clinical Genomic Science (Wallis et al., 2013). Classification of CNVs was performed according to the 5-class system (Class 1–5) of the European guidelines for constitutional cytogenomic analysis (Silva et al., 2019). Of note, variants identified would only be validated using orthogonal methods if quality control metrics and/or visual inspection of the BAM files indicated this need. Upon the completion of variant interpretation, the laboratory specialist clinical genetics immediately communicated the results to the requesting physician. Simultaneously, a written report was made available via regular secured mail or through the electronic healthcare system.

The diagnostic outcome was either one of the following three options:

i) A conclusive diagnosis is reached: a (likely) pathogenic variant (class 4 or 5) is identified in a known disease gene which explains the patients’ phenotype.
ii) A possible diagnosis is obtained: a variant of unknown significance (class 3) is identified in a known gene which may explain the patient’s phenotype, or alternatively, a (likely) pathogenic variant (class 4 or 5) is identified in a candidate disease gene.
iii) A negative report: no variants (class 3, 4 or 5) were identified that may explain the patient’s phenotype.

Of note, in case of the identification of an unsolicited finding, referring to the identification of a (likely) pathogenic variant (class 4 or 5) in a gene unrelated to the patient’s phenotype but of clinical relevance, a routine procedure followed, involving the instant discussion of the variant in a committee advising on its disclosure (van der Schoot et al., 2022).

### Statistical methods

Data analysis of the clinical variables was performed in Excel (version 2016). In more detail, normal distributed data were expressed in mean and standard deviation; Median and interquartile ranges were used in data with a skewed distribution. For categorical data statistical analysis was performed using descriptive and chi-square analysis, and for continuous variables a two-sided Fisher’s exact test was used.

## Results

### Characteristics of the rES procedure

Between Jan-2016 and Dec-2019, rES was performed for 575 critically ill patients in whom a genetic disorder was suspected (Table 1; Supplementary Table S1). Upon the first introduction of rES, only very few patients were sequenced (n = 2 in all of Q1-2016), whereas this rapidly increased to ∼5/week (from Q1-2018 onwards) and subsequently to ∼10 each week (Q3-2019 onwards; Supplementary Figure S1). In fact, more than half (n = 322, 56%) of the cohort received rES in 2019. With a marked increase in the number of rES tests conducted per time interval, there was a reduction in the median turnaround time (TAT) from 17 days (IQR 14–19 days) in Q1-2016 to 11 days (IQR 8–15 days) in Q4-2019. The latter has been a direct consequence of optimization of the rES process, including further automation of the library preparation as well as performing multiple sequence runs per week (Supplementary Figure S2).

**TABLE 1:** Characteristics patient cohort.

	*Total cohort n = 575*
Prenatal/Postnatal	241 (42%)/334 (58%)
Male/Female (postnatal only)	175 (52%)/159(48%)
Median gestational age prenatal samples	20 weeks (IQR 20–21)
*Age group*	
Prenatal	241 (42%)
Neonatal (0–4 weeks)	114 (20%)
Infants (1–23 months)	115 (20%)
Child, preschool (2–5 years)	36 (6%)
Child (6–12 years)	20 (4%)
Adolescence (13–18 years)	13 (2%)
Adult (19–65 years)	36 (6%)
*Sequencing strategy*	
Singleton	80 (14%)
Duo	1 (<1%)
Trio	491 (85%)
Quartet	3 (<1%)

#### Diagnostic strategies for patients receiving rES

Trio sequencing was performed for 85% of the patients (n = 491), 14% (n = 80) was sequenced as singleton, and the remainder either as part of a duo (n = 1, <1%) or quartet (n = 3, <1%) (Table 1; Supplementary Table S1). To gain insight into the phase of life rES was performed, we categorized patients into seven age groups: prenatal (before birth), neonatal (0–4 weeks), infancy (1–23 months), preschool (2–5 years), childhood (6–12 years), adolescence (13–18 years), and adulthood (19 years and above). The majority of rES was performed in the prenatal phase (42%), followed by newborns (20%) and infants (20%) (Table 1; Supplementary Table S1).

**FIGURE 1:**
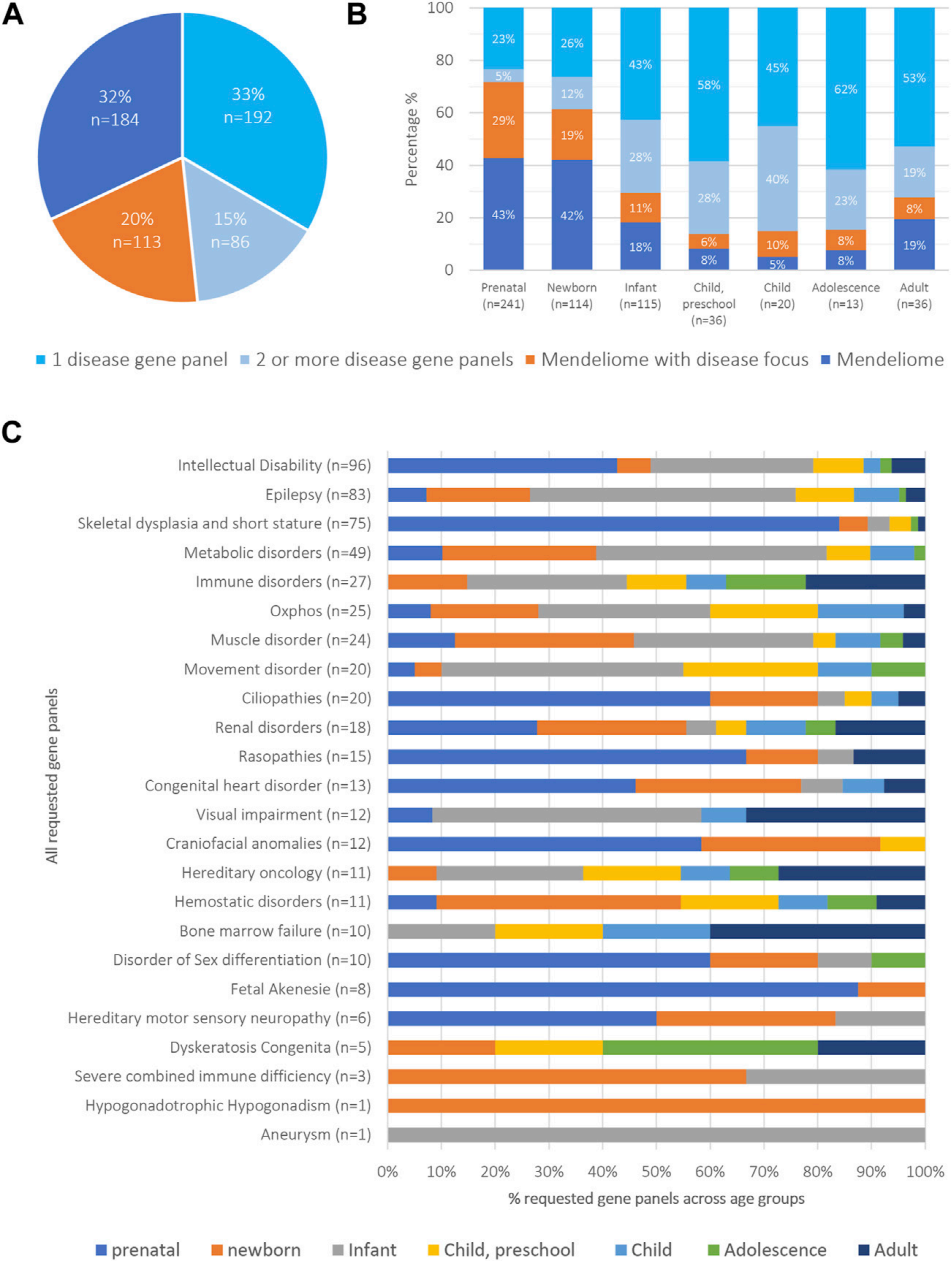
Overview of cohort categorized by diagnostic strategies and age groups **(A)** Diagnostic strategies were classified into four categories: i) specific phenotypic presentation requiring the analysis of a single disease-gene panel, applied to 15% of patients; ii) clinical spectrum requiring the analysis of multiple disease-gene panels (33% of patients); iii) Mendeliome analysis with a specific emphasis on certain disease categories (20%), and iv) Mendeliome without a clear disease focus because of too non-specific and/or too broad phenotypic presentation (32%). **(B)** Distribution of diagnostic strategies across the different age groups. **(C)** Total number of requested gene panels and their distribution across different age groups.

Based on the clinical presentation of the patients, the clinicians choose a targeted diagnostic strategy for 278 patients (48%), involving the analysis of variants restricted to one (n = 192; 33%) or multiple disease-gene panels (n = 86; 15%, range of panels between 2 and 5) (Figure 1A). For 113 other patients (20%), the strategy involved analysis of variants in the Mendeliome (3,839 genes known to be involved in disease) plus one or more disease gene panels with particular focus to certain disease categories. For the remaining 184 patients (32%), the phenotype did not allow to select for a disease focus (e.g., too broad or too unspecific), resulting in analysis of the Mendeliome, without any other specific focus areas (Figure 1A). Interestingly, the diagnostic strategy of requesting rES varied not only among the age groups, with analysis of the Mendeliome (with or without addition of disease-gene panels) being more often requested in a prenatal setting and neonates (Figure 1B), but also the type of disease-gene panels tested across the various age groups showed marked differences (Figure 1C), with analysis related to craniofacial disorders restricted to prenatal requests until preschool age, whereas analysis for bone marrow failure/cancer was mostly observed in adolescence and adults.

#### Diagnostic yield

**FIGURE 2:**
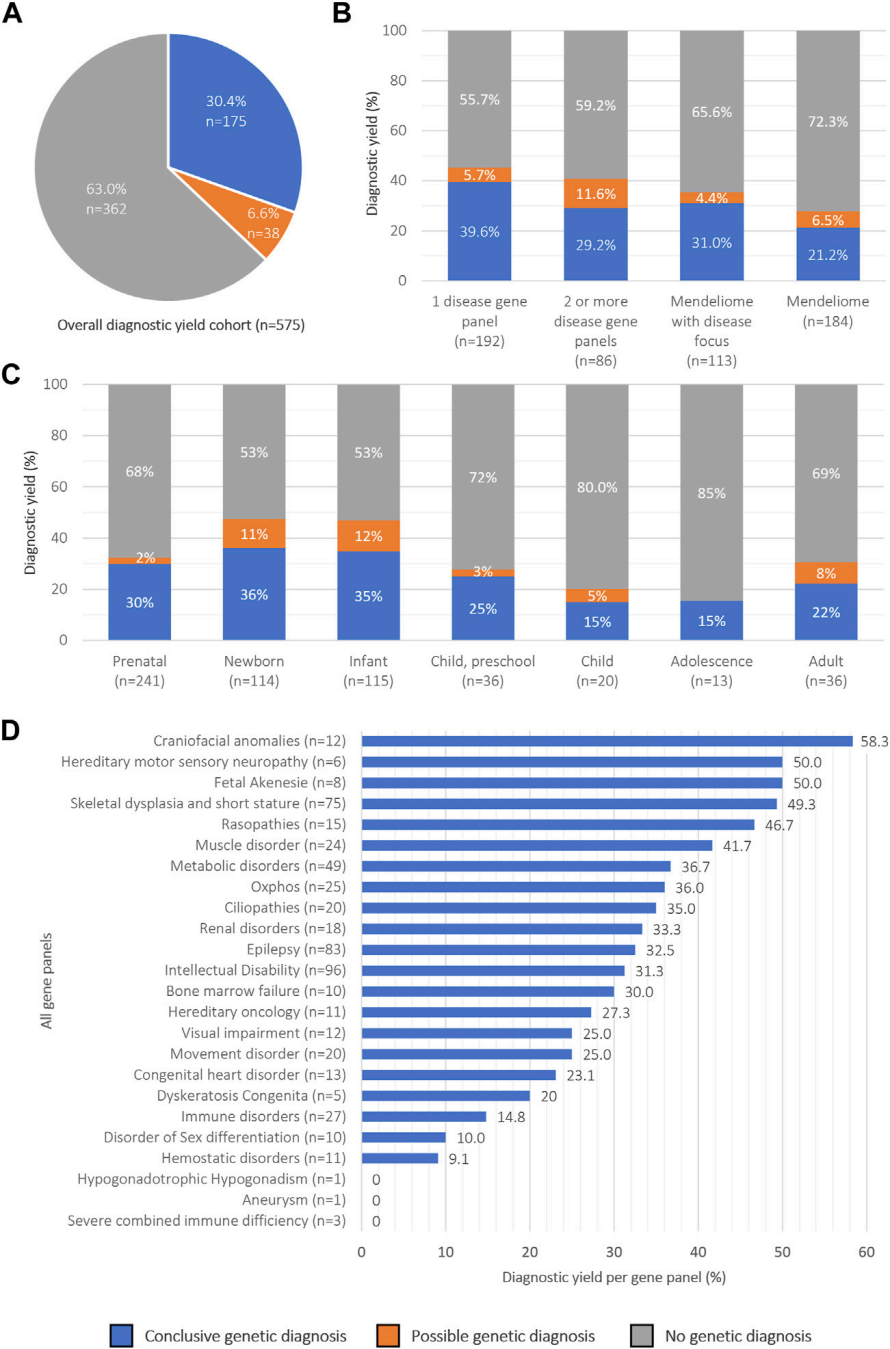
Overview of rES diagnostic yield categorized by diagnostic strategies and age groups **(A)** Pie chart showing the overall percentage of diagnostic yield obtained in 575 patients. **(B)** Diagnostic success rates across diagnostic strategies, **(C)** age groups and **(D)** disease gene panels.

The overall diagnostic yield was 30.4% (in 175 of 575 individuals) across the different age groups and diagnostic strategies (Figure 2A). For 116 of them, a dominant disease was identified, in 47 a recessive, and in 11 an X-linked disorder; for one additional individual a dual diagnosis was established, consisting of a dominant and a recessive disease (Table 2). For 156 (89.1%) individuals, the causal variant(s) were SNV/indels, whereas CNVs were observed in 18 (10.3%) (Supplementary Table S2). For one individual (<1%), the recessive disorder was caused by the combination of an SNV + CNV. In addition to a conclusive diagnosis, 6.6% of individuals received a possible diagnosis (n = 38; Supplementary Table S3); in the remainder of individuals (63.0%, n = 362), a genetic cause remained elusive.

**TABLE 2:** Inheritance and variant type distribution for individuals with a conclusive diagnosis.

	Individuals (n = 175)*
**Dominant**	117^a^
*De novo*	*99*
*Inherited*	*11*
*Unknown*	*7*
**Recessive**	**48** ^b^
*Homozygous*	*29*
*Compound heterozygous*	*19*
**X-linked**	**11**
*All types*	*11*

^a^ *Details of variants are provided in* Supplementary Table S2.

^b^ *One individual received a dual diagnosis, of which one was a homozygous variant for a recessive disorder, and one a de novo variant for a dominant disease*.

The bold values represent the three main categories used for classifying the inheritance and variant type distribution of individuals with a conclusive diagnosis.

We then proceeded to evaluate potential variances in diagnostic yield across different diagnostic strategies and age groups (Figures 2B, C). Regarding diagnostic strategies, interpretation of rES data guided by a well-described clinical presentation (n = 391) did yield significantly more diagnoses (34.8%; n = 136) than interpretation of the Mendeliome (n = 184) without a clear clinical lead (21.2%; n = 39; Fisher’s Exact *p* = 0.003) (Figure 2B). The results indicated that the diagnostic yield was the highest in newborns (36%), and lowest in children (15%) and adolescents (15%), although not statistically different (Fisher’s exact *p* = 0.08 and *p* = 0.22, respectively Figure 2C). Additionally, there was no statistically significant difference in diagnostic yields when the cohort was split in individuals under 18 years of age (31.0%) and those aged 19 years and older (22.2%) (Fisher’s exact, *p* = 0.35). To determine whether specific clinical indications yielded a higher diagnostic yield than others, we subsequently analyzed the yields across panels (Figure 2D). In total 555 disease-specific panels (not including the Mendeliome) were analyzed in 391 individuals (average 1.4 per individual), of which intellectual disability (n = 96) and epilepsy (n = 83) were most frequently requested. Diagnostic yields across the panels varied between 0% (e.g., Severe combined immune deficiency, requested 3 times) and 58.3% for craniofacial anomalies (12 times requested).

### Unsolicited findings

In addition to disease-causing variants, three unsolicited findings (UF) were identified in this cohort (0.52%) (Supplementary Table S4). This incidence is similar to the incidence of 0.58% previously reported in routine postnatal care based on disease-gene panel strategies, as well as when corrected for the interpretation of the Mendeliome (1.01% in this cohort, and 1.03% in literature, Fisher’s exact test *p* = 1.0) (Wallis et al., 2013).

## Discussion

Research on the clinical utility of rES has so far primarily focused on the value of rES for fetuses, neonates, and infants in different ICU settings, and has shown great successes in establishing genetic diagnoses and impacting clinical decision-making (Meng et al., 2017; Stark et al., 2018; Sanford et al., 2019). The evidence for the clinical utility of rES for older children and adult patients is, however, limited. We therefore retrospectively examined the outcomes of rES of 575 critically ill patients over a 4-year time period. All these patients received rES as it was expected that genetic insight into their disease would help in clinical decision-making. Our results demonstrate that rES is useful for all critically ill patients across all ages equally, and across the broad spectrum of rare disease.

The overall diagnostic yield achieved in our study was 30.4%, which is comparable to the yield of 20%–45% described in previous studies performed, albeit that these mostly focused on critically ill neonates, infants and prenatal samples (Kingsmore et al., 2019; Sanford et al., 2019; Deden et al., 2020; McDermott et al., 2022; Faas et al., 2023). Based on the inclusion of different age groups, we were able to determine the diagnostic range, which varied between 15% (for children/adolescents) to 35% (for newborns). Although the individual groups vary in size, we noted that there were no statistical differences in diagnostic yield across these different age groups. So far, few studies have been performed on adults receiving rES, also limiting the possibilities for formal comparisons. One such study, however, comparing adult rES outcomes to pediatric rES also noted that there is no difference in diagnostic yield between these groups (Kamolvisit et al., 2021). Of note, the overall diagnostic yield of 57% for adults in this published study (for 4/7 patients) was higher than obtained in our cohort (22%, for 8/36). Whereas this can be explained by the small numbers, it might also be explained by the different (adult) phenotypes investigated and underlying diagnostic strategies impacting diagnostic yield as well as a more stringent clinical pre-selection for the likelihood of the disease being of genetic origin.

In addition to 30.4% conclusive diagnosis, we also observed a possible diagnosis in 6.6% of patients. Interestingly, this percentage seems lower than possible diagnosis reported in exome sequencing studies performed in a non-rapid situation. For instance, for neurodevelopmental disorders, possible diagnoses often reflect (*de novo*) variants in genes without an established disease-gene association (e.g., candidate disease gene) (de Ligt et al., 2012; Vissers et al., 2017; Wright et al., 2023). The latter may suggest that when performing exome sequencing in a rapid scenario, there is a stronger focus on reporting variants in genes with known disease-gene associations, resulting in less variants of unknown clinical significance, and less reports of candidate disease-genes. This hypothesis would make sense as the rapid exomes are performed to inform clinical decision making, which notably can only be made on the basis of (likely) pathogenic variants in genes with well-established genotype-phenotype associations.

In our retrospective analysis, we conducted various subgroup analyses and substantiated that clinical pre-selection, typically based on phenotype, along with differing diagnostic strategies, significantly impact diagnostic yield. For instance, in a prenatal setting, congenital malformations visible upon ultrasounds provide a strong suspicion for a genetic disorder and provide an overall high diagnostic yield (Deden et al., 2020; Slavotinek et al., 2023). When combined with a clinical phenotype providing clues towards the origin, such as for instance “craniofacial anomalies”, we noted that the diagnostic yield raised to 58.3%, despite the large genetic heterogeneity of the associated phenotypes. Contrastingly, we also confirmed the observation previously made by us and others that perinatal phenotypes were often non-specific or too broad to hint towards certain clinical entities, as could be observed by the frequent request of the Mendeliome without additional specification: the Mendeliome was requested 43% for prenatal and neonatal cases, in contrast to only 5%–19% of the other age groups (Normand et al., 2018; Deden et al., 2020). In line with the reasoning that clinical characteristics may become more recognizable and/or specific is substantiated by our data that with increasing age, more often disease-specific gene panels were requested, although the panels differed among age groups, focusing on those diseases most acutely presenting during the respective phase of life.

The overall diagnostic yield does thus not differ from those obtained in non-acute settings for the respective diseases (Normand et al., 2018; Corsten-Janssen et al., 2020; Deden et al., 2020), suggesting that the main and only difference is the time from clinical referral to diagnosis, which is especially important in clinically critical situations as a prompt diagnosis will usually result in a quicker initiation of optimal treatment. In our hands, the median rES turnaround time is 11 days (range 8–15 days) which is within the general range from previous publications for rES studies (Saunders et al., 2012; Kingsmore et al., 2019; Sanford et al., 2019), and significantly shorter compared to standard ES analyses (136 days) (Stark et al., 2018). To stabilize this turnaround time despite the increasing number of diagnostic requests, we optimized the workflows by increasing sequencing throughput, automation of laboratory work, and prioritization of data in the bioinformatic pipelines and clinical interpretation. In recent years, there has been a significant increase in demand for rES, resulting in substantial growth in numbers and further solidifying the workflows within our center. Others have already shown examples of 4 days from request to report, underscoring that a faster time-to-diagnosis might still be feasible, albeit to be determined at what scale further technical and infrastructure optimization is required to perform this at a large scale (Vissers et al., 2010; Kingsmore et al., 2019). One obvious choice might be the step from rES to rapid genomes, as this would overcome the time lost for targeted enrichment of the exome (Clark et al., 2019; Kingsmore et al., 2019; Sanford et al., 2019).

The timing of rES is essential, especially for situations where clinical decision-making can be impacted by knowledge on the genetic underlying cause (Powis et al., 2020). Compelling evidence on impacting medical management have been shown in neonatal situations (Meng et al., 2017; Freed et al., 2020; Kamolvisit et al., 2021; Krantz et al., 2021; McDermott et al., 2022), but less examples are presented on the adult population, although the impact on the patient’s outcome is similar. For example, a male adult suffering from myelofibrosis and autoinflammatory symptoms underwent rES, with results available within 9 days. The analysis identified a somatic variant in *MPL* (related to myelofibrosis) and a heterozygous variant in *ACP5* (associated with immune dysregulation disorder), both matching the patient’s phenotype. Based on these findings, the patient’s medication was modified, resulting in a significant improvement in the patient’s condition. Another interesting observation for the use of rES in the adult population involved the requests for the disease-gene panel associated with intellectual disability, given the few possibilities for medical interventions upon identification of the cause of disease. More detailed evaluation revealed that these requests mostly dealt with (ongoing) pregnancies of a blood relative of the index, and were thus not performed to impact the medical decisions of the index but that of direct family members (data not shown). The fact that rES was now possible allowed these siblings to make better informed decisions on reproductive options. Of note, although rES is primarily performed to find the genetic cause of disease, even when rES does not lead to a specific genetic diagnosis, it can still impact clinical management by allowing for the continuation (or discontinuation) of intensive therapy in the absence of a lethal condition (Duyzend, 2020), end-of-life decisions (Krantz et al., 2021), and/or perinatal choices (Deden et al., 2020).

Ethical concerns have been raised with regard to performing rES for patients in relation to unsolicited findings. On one hand, these include pre-test counseling and the patients being able to make an informed decision with respect to understanding the risk of unsolicited findings, given the time and emotional pressure inflicted by the critical medical situation (Olde Keizer et al., 2023). On the other hand, the unsolicited findings relate to possible filtering and prioritization strategies of genetic variants and the impact on the identification of UFs. With regards to the time and emotional pressure, we recently reported that parents of children admitted to the neonatal intensive care unit who underwent rES seemingly experienced no increased distress resulting from the time pressure when opting for rES (Olde Keizer et al., 2023), and the risk on UFs. Little is, however, known on the incidence of UFs in rES when compared to “regular ES”, for which reports have shown that the incidence of UFs varies between 0.04% and 1.03%, depending on diagnostic strategies (gene panel(s) vs Mendeliome, respectively) (van der Schoot et al., 2022). Our retrospective analysis now contributes two additional observations. Firstly, our analyses show that approximately half of all rES is performed using disease-gene panel strategies, thus limiting the risk on UFs. Secondly, in our cohort, only three UFs have been disclosed, suggesting that–after correction for diagnostic strategy–the incidence of UFs in rES is equal to the incidence of UFs in a routine postnatal ES setting.

In conclusion, this study highlights the efficacy of rES as a first-tier genetic test for critically ill patients, encompassing the full range of rare diseases, and more widely generalizable to clinical practice beyond (neonatal/pediatric) intensive care units, and with similar clinical importance across all age groups.

## Data Availability

The original contributions presented in the study are included in the Supplementary Material, further inquiries can be directed to the corresponding author.

## References

[B1] Clark M. M. Hildreth A. Batalov S. Ding Y. Chowdhury S. Watkins K. (2019). Diagnosis of genetic diseases in seriously ill children by rapid whole-genome sequencing and automated phenotyping and interpretation. Sci. Transl. Med. 11 (489), eaat6177. 10.1126/scitranslmed.aat6177 31019026 PMC9512059

[B2] Corsten-Janssen N. Bouman K. Diphoorn J. C. D. Scheper A. J. Kinds R. el Mecky J. (2020). A prospective study on rapid exome sequencing as a diagnostic test for multiple congenital anomalies on fetal ultrasound. Prenat. Diag 40 (10), 1300–1309. 10.1002/pd.5781 PMC7540374 32627857

[B3] Deden C. Neveling K. Zafeiropopoulou D. Gilissen C. Pfundt R. Rinne T. (2020). Rapid whole exome sequencing in pregnancies to identify the underlying genetic cause in fetuses with congenital anomalies detected by ultrasound imaging. Prenat. Diag 40 (8), 972–983. 10.1002/pd.5717 PMC7497059 32333414

[B4] de Ligt J. Willemsen M. H. van Bon B. W. Kleefstra T. Yntema H. G. Kroes T. (2012). Diagnostic exome sequencing in persons with severe intellectual disability. N. Engl. J. Med. 367, 1921–1929. 10.1056/NEJMoa1206524 23033978

[B5] D’Gama A. M. Del Rosario M. C. Bresnahan M. A. Yu T. W. Wojcik M. H. Agrawal P. B. (2022). Integrating rapid exome sequencing into NICU clinical care after a pilot research study. Npj Genomic Med. 7 (1), 51. 10.1038/s41525-022-00326-9 PMC9441819 36064943

[B6] Diekstra A. Bosgoed E. Rikken A. van Lier B. Kamsteeg E. J. Tychon M. (2015). Translating sanger-based routine DNA diagnostics into generic massive parallel ion semiconductor sequencing. Clin. Chem. 61 (1), 154–162. 10.1373/clinchem.2014.225250 25274553

[B7] Duyzend M. H. (2020). Non-diagnostic results from rapid exome sequencing can change clinical management in the critical care setting. J. Pediatr. 226, 1–4. 10.1016/j.jpeds.2020.09.023

[B8] Dye D. E. Brameld K. J. Maxwell S. Goldblatt J. O'Leary P. (2011). The impact of single gene and chromosomal disorders on hospital admissions in an adult population. J. Commun. Genet. 2 (2), 81–90. 10.1007/s12687-011-0043-3 PMC3186028 22109792

[B9] Faas B. H. W. Westra D. de Munnik S. A. van Rij M. Marcelis C. Joosten S. (2023). All-in-one whole exome sequencing strategy with simultaneous copy number variant, single nucleotide variant and absence-of-heterozygosity analysis in fetuses with structural ultrasound anomalies: a 1-year experience. Prenat. Diagn 43 (4), 527–543. 10.1002/pd.6314 36647814

[B10] Freed A. S. Clowes Candadai S. V. Sikes M. C. Thies J. Byers H. M. Dines J. N. (2020). The impact of rapid exome sequencing on medical management of critically ill children. J. Pediatr. 226, 202–212.e1. 10.1016/j.jpeds.2020.06.020 32553838 PMC7736066

[B11] Kamolvisit W. Phowthongkum P. Boonsimma P. Kuptanon C. Rojnueangnit K. Wattanasirichaigoon D. (2021). Rapid exome sequencing as the first-tier investigation for diagnosis of acutely and severely ill children and adults in Thailand. Clin. Genet. 100 (1), 100–105. 10.1111/cge.13963 33822359

[B12] Kingsmore S. F. Cakici J. A. Clark M. M. Gaughran M. Feddock M. Batalov S. (2019). A randomized, controlled trial of the analytic and diagnostic performance of singleton and trio, rapid genome and exome sequencing in ill infants. Am. J. Hum. Genet. 105 (4), 719–733. 10.1016/j.ajhg.2019.08.009 31564432 PMC6817534

[B13] Krantz I. D. Medne L. Weatherly J. M. Wild T. Biswas S. Devkota B. (2021). Effect of whole-genome sequencing on the clinical management of acutely ill infants with suspected genetic disease A randomized clinical trial. Jama Pediatr. 175 (12), 1218–1226. 10.1001/jamapediatrics.2021.3496 34570182 PMC8477301

[B14] Lord J. McMullan D. J. Eberhardt R. Y. Rinck G. Hamilton S. J. Quinlan-Jones E. (2019). Prenatal exome sequencing analysis in fetal structural anomalies detected by ultrasonography (PAGE): a cohort study. Lancet 393 (10173), 747–757. 10.1016/S0140-6736(18)31940-8 30712880 PMC6386638

[B15] McDermott H. Sherlaw-Sturrock C. Baptista J. Hartles-Spencer L. Naik S. (2022). Rapid exome sequencing in critically ill children impacts acute and long-term management of patients and their families: a retrospective regional evaluation. Eur. J. Med. Genet. 65 (9), 104571. 10.1016/j.ejmg.2022.104571 35842091

[B16] Meng L. Y. Pammi M. Saronwala A. Magoulas P. Ghazi A. R. Vetrini F. (2017). Use of exome sequencing for infants in intensive care units ascertainment of severe single-gene disorders and effect on medical management. Jama Pediatr. 171 (12), e173438. 10.1001/jamapediatrics.2017.3438 28973083 PMC6359927

[B17] Normand E. A. Braxton A. Nassef S. Ward P. A. Vetrini F. He W. (2018). Clinical exome sequencing for fetuses with ultrasound abnormalities and a suspected Mendelian disorder. Genome Med. 10 (1), 74. 10.1186/s13073-018-0582-x 30266093 PMC6162951

[B18] Olde Keizer R. Marouane A. Kerstjens-Frederikse W. S. Deden A. C. Lichtenbelt K. D. Jonckers T. (2023). Rapid exome sequencing as a first-tier test in neonates with suspected genetic disorder: results of a prospective multicenter clinical utility study in The Netherlands. Eur. J. Pediatr. 182, 2683–2692. 10.1007/s00431-023-04909-1 36997769 PMC10257607

[B19] Powis Z. Hagman K. D. F. Blanco K. Au M. Graham J. M. Singh K. (2020). When moments matter: finding answers with rapid exome sequencing. Mol. Genet. Genom Med. 8 (2), e1027. 10.1002/mgg3.1027 PMC7005623 31872981

[B20] Radboudumc (2023). Exome sequencing diagnostics: exome panels. Avaliable at: https://www.radboudumc.nl/en/patient-care/patient-examinations/exome-sequencing-diagnostics/information-for-referrers/exome-panels .

[B21] Sanford E. F. Clark M. M. Farnaes L. Williams M. R. Perry J. C. Ingulli E. G. (2019). Rapid whole genome sequencing has clinical utility in children in the PICU. Pediatr. Crit. Care Me 20 (11), 1007–1020. 10.1097/PCC.0000000000002056 PMC6832787 31246743

[B22] Saunders C. J. Miller N. A. Soden S. E. Dinwiddie D. L. Noll A. Alnadi N. A. (2012). Rapid whole-genome sequencing for genetic disease diagnosis in neonatal intensive care units. Sci. Transl. Med. 4 (154), 154ra135. 10.1126/scitranslmed.3004041 PMC4283791 23035047

[B23] Silva M. de Leeuw N. Mann K. Schuring-Blom H. Morgan S. Giardino D. (2019). European guidelines for constitutional cytogenomic analysis. Eur. J. Hum. Genet. 27 (1), 1–16. 10.1038/s41431-018-0244-x 30275486 PMC6303289

[B24] Slavotinek A. Rego S. Sahin-Hodoglugil N. Kvale M. Lianoglou B. Yip T. (2023). Diagnostic yield of pediatric and prenatal exome sequencing in a diverse population. NPJ Genom Med. 8 (1), 10. 10.1038/s41525-023-00353-0 37236975 PMC10220040

[B25] Stark Z. Lunke S. Brett G. R. Tan N. B. Stapleton R. Kumble S. (2018). Meeting the challenges of implementing rapid genomic testing in acute pediatric care. Genet. Med. 20 (12), 1554–1563. 10.1038/gim.2018.37 29543227

[B26] van der Schoot V. Haer-Wigman L. Feenstra I. Tammer F. Oerlemans A. J. M. van Koolwijk M. P. A. (2022). Lessons learned from unsolicited findings in clinical exome sequencing of 16,482 individuals. Eur. J. Hum. Genet. 30 (2), 170–177. 10.1038/s41431-021-00964-0 34697415 PMC8821629

[B27] Vissers L. van Nimwegen K. J. M. Schieving J. H. Kamsteeg E. J. Kleefstra T. Yntema H. G. (2017). A clinical utility study of exome sequencing versus conventional genetic testing in pediatric neurology. Genet. Med. 19 (9), 1055–1063. 10.1038/gim.2017.1 28333917 PMC5589982

[B28] Vissers LELM de Ligt J. Gilissen C. Janssen I. Steehouwer M. de Vries P. (2010). A *de novo* paradigm for mental retardation. Nat. Genet. 42, 1109–1112. 10.1038/ng.712 21076407

[B29] Wallis Y. Payne S. McAnulty C. Bodmer D. Sistermans E. Robertson K. (2013). Practice guidelines for the evaluation of pathogenicity and the reporting of sequence variants in clinical molecular genetics. Association for Clinical Genetic Science & Dutch Society of Clinical Genetic Laboratory Specialists, 1–16.

[B30] Wright C. F. Campbell P. Eberhardt R. Y. Aitken S. Perrett D. Brent S. (2023). Genomic diagnosis of rare pediatric disease in the United Kingdom and Ireland. New Engl. J. Med. 388 (17), 1559–1571. 10.1056/NEJMoa2209046 37043637 PMC7614484

[B31] Wu E. T. Hwu W. L. Chien Y. H. Hsu C. Chen T. F. Chen N. Q. (2019). Critical trio exome benefits in-time decision-making for pediatric patients with severe illnesses. Pediatr. Crit. Care Me 20 (11), 1021–1026. 10.1097/PCC.0000000000002068 31261230

[B32] Yang Y. Muzny D. M. Reid J. G. Bainbridge M. N. Willis A. Ward P. (2013). Clinical whole-exome sequencing for the diagnosis of Mendelian disorders. New Engl. J. Med. 369, 1502–1511. 10.1056/NEJMoa1306555 24088041 PMC4211433

