# Supplementary material for: Lessons learned from rapid exome sequencing for 575 critically ill patients across the broad spectrum of rare disease
Source: Front Genet. 2024 Jan 8;14:1304520. doi: 10.3389/fgene.2023.1304520 (PMC10800954; doi:10.3389/fgene.2023.1304520)
Supplement: Supplementary file 4 [file DataSheet1.DOCX]

**Supplementary material**

Supplementary material include 2 figures and 4 tables

Figure S1. Schematic representation of turnaround time (TAT) of rES combined with the number of rES performed in a given time period

Figure S2. Schematic representation of rES procedure

Table S1. Clinical descriptions of cohort

Table S2. Variant details of conclusive diagnoses

Table S3. Variant details of possible diagnoses

Table S4. Unsolicited findings

**Supplementary Figure 1 |** Schematic representation of turnaround time (TAT) of rES combined with the number of rES performed in a given time period


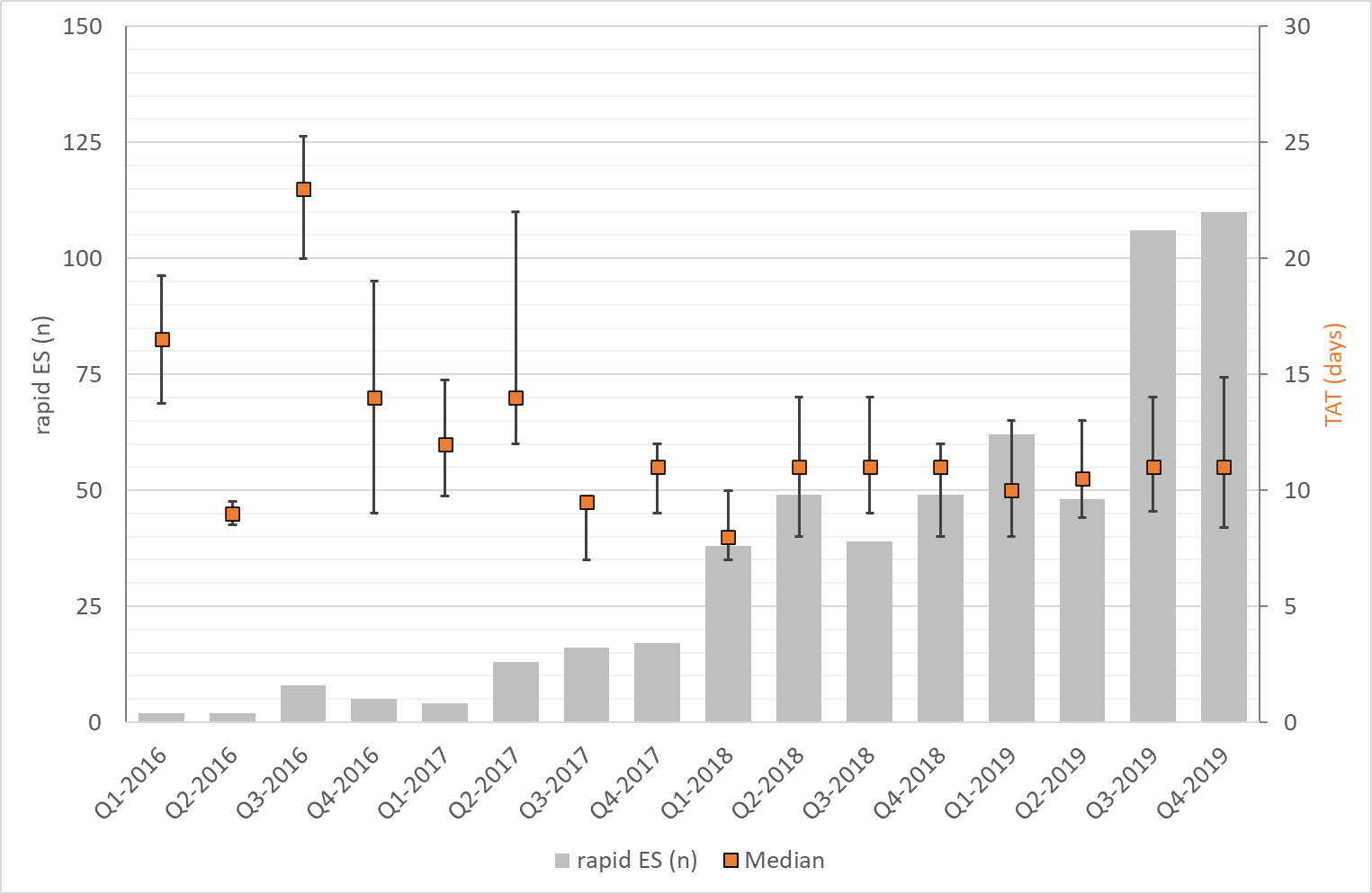


**Supplementary Figure 1**  | **Schematic representation of turnaround time (TAT) of rES combined with the number of rES performed in a given time period.** Early 2016, only few rES tests were performed, with a median TAT of 16 days. Whereas over time the number of rES test performed per time interval increased significantly to up to 110 requests in Q4-2019, the median TAT reduced to 11 days, and has remained stable since Q3-2017 despite the increase in request. The latter has been a direct consequence of a higher number of requests, a herewith required change of the sequencing platform, and automation of the process (see also Figure S2). Please note that the number of samples sequenced only displays index patients, and that it does not include accompanying parental samples.

**Supplementary Figure 2 |** Schematic representation of rES procedure

**
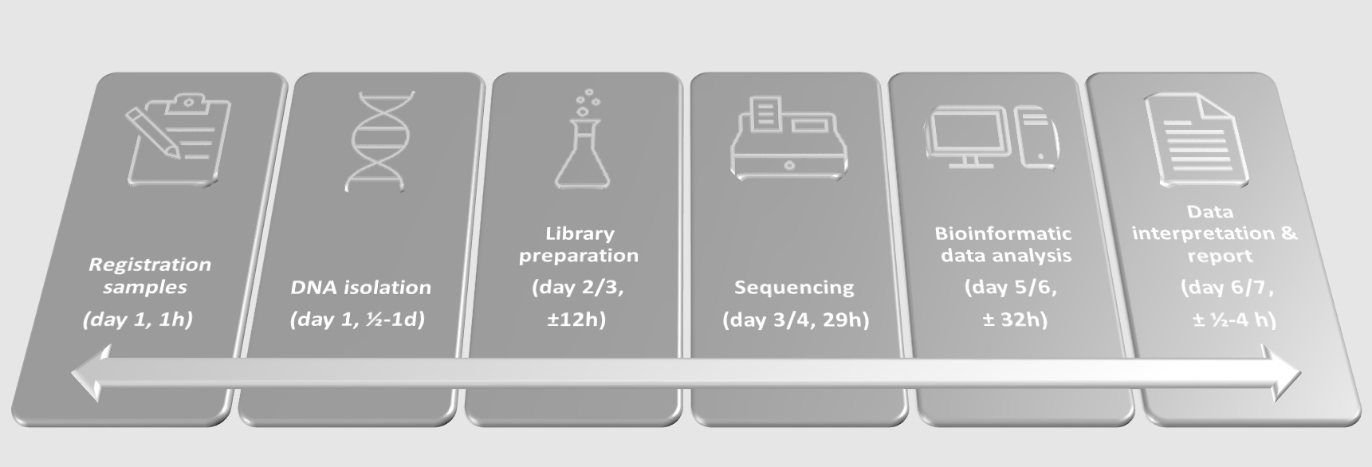
**

11 days (IQR 8-15 days)

**Supplementary Figure 2. Schematic presentation of the rES workflow using the NextSeq 500, as implemented originally in 2016.** For rapid WES, usually EDTA blood samples (or occasionally previously isolated DNAs) were analyzed. All samples were registered at the sample reception (˜1 hour) before being transferred to the DNA isolation facility, where DNA was isolated in an automated fashion (0.5-1 day, if necessary). Initially, library preparation was initiated on a weekly basis, primarily commencing on Mondays. This process required approximately 12 working hours, distributed over two days, and involved an overnight hybridization step. Accordingly, the sequencer was started the next day, and was running approximately 29 hours. Once the sequencing run was (automatically) detected to be finished, an automated bioinformatic pipeline started demultiplexing, alignment and subsequent variant calling and annotation. Following an automated quality control (QC), data was transferred automatically to a team of laboratory specialists clinical genetics for interpretation (˜0.5-4 hours). In the most optimal scenario, the whole process needed around 6-7 working days. Modification of the process for optimization of throughput included replacing the NextSeq 500 for a NovaSeq6000, automation of the library preparation, and starting of the workflow multiple times a week. Accordingly, turnaround times could be reduced from a median of 17 to a median of 11 days, despite of growing numbers of analyzed samples (see also Figure S1).

**Supplementary Table 1 |** Clinical description of the cohort

*[Online Excel table]*

**Supplementary Table 2 |** Variant details of conclusive diagnoses

*[Online Excel table]*

Tab 1: Details of all SNV and indels leading to conclusive genetic diagnosis

Tab 2: Details of all CNVs leading to conclusive genetic diagnosis

**Supplementary Table 3 |** Variant details of possible diagnoses

*[Online Excel table]*

Tab 1: Details of all SNV and indels leading to possible genetic diagnosis

Tab 2: Details of all CNVs leading to possible genetic diagnosis

**Supplementary Table 4 |** Unsolicited findings

| Gene | Variant (genomic position) | cDNA | Protein | Zygosity and Inheritance | Associated phenotype |
| --- | --- | --- | --- | --- | --- |
| *PKD2* | Chr4(GRCh37):g.88996055C>T | NM_000297.3: c.2614C>T | p.(Arg872*) | Heterozygous, paternal | Polycystic kidney disease 2 |
| *MYBPC3* | Chr11(GRCh37):9.47362755C>T | NM_000256.3: c.1831G>A | p.(Glu611Lys) | Heterozygous, paternal | Dilated cardiomyopathy |
| *PKP2* | Chr12(GRCh37): g.33049611G>A | NM_004572.3: c.55C>T | p.(Gln19*) | Heterozygous, maternal | Arrhythmogenic right ventricular cardiomyopathy |
